# The Influence of SARS-CoV-2 Infection on Acute Myocardial Infarction Outcomes

**DOI:** 10.3390/jcm12185899

**Published:** 2023-09-11

**Authors:** Eugeniusz Hrycek, Anna Walawska-Hrycek, Maciej Hamankiewicz, Krzysztof Milewski, Przemysław Nowakowski, Piotr Buszman, Aleksander Żurakowski

**Affiliations:** 1 American Heart of Poland, Topolowa 16, 32-500 Chrzanów, Poland; 2 Department of Cardiology, Faculty of Medical Sciences, Andrzej Frycz Modrzewski Kraków University, 30-705 Kraków, Poland; 3 Department of Neurology, Faculty of Medical Sciences in Katowice, Medical University of Silesia, 40-752 Katowice, Poland; 4 American Heart of Poland, Warszawska 52, 40-008 Katowice, Poland; 5 American Heart of Poland, Armii Krajowej 101, 43-316 Bielsko-Biała, Poland; 6 Department of Vascular Surgery, Faculty of Medical Sciences, University of Technology, Rolna 43, 40-555 Katowice, Poland

**Keywords:** COVID-19, heart failure, myocardial infarction, respiratory failure

## Abstract

Background: This multicenter retrospective study with a control group was designed to assess the influence of severe acute respiratory syndrome coronavirus 2 (SARS-CoV-2) infection on the outcomes of patients with myocardial infarction (MI). Methods: A total of 129 patients with COVID-19 who were treated for MI were included in this study. The control group comprised 129 comparable patients without SARS-CoV-2 infection. The in-hospital, out-of-hospital, and overall mortality were analyzed. Results: A total of thirty-one (24%) patients died in the study group, and two (1.6%) patients died in the control group (OR = 20.09; CI: 4.69–85.97; *p* < 0.001). Similar results were observed in all analyzed patient subgroups. Multivariable Cox regression analysis confirmed the significant influence of SARS-CoV-2 infection on in-hospital outcomes (HR: 8.48459; CI: 1.982–36.320; *p* = 0.004). Subanalysis of the groups with COVID-19 plus ST-elevation MI (STEMI) or non-ST-elevation MI (NSTEMI) revealed comparable mortality rates: 14 (21.12%) patients in the NSTEMI group and 17 (26.98%) patients in the STEMI subgroup died (OR: 1.3; CI: 0.56–3.37; *p* = 0.45). During out-of-hospital observation, no differences in mortality were observed (OR: 0.77; CI: 0.11–4.07; *p* = 0.73). Conclusions: SARS-CoV-2 infection affects the in-hospital outcomes of patients with both MI and COVID-19, regardless of MI type (STEMI vs. NSTEMI).

## 1. Introduction

Since 2019, coronavirus disease 2019 (COVID-19) has become an overwhelming and growing challenge for healthcare systems. This disease is caused by severe acute respiratory syndrome coronavirus 2 (SARS-CoV-2), a single-stranded RNA virus that is a member of the coronavirus family and was initially identified in Wuhan, China. SARS-CoV-2 enters cells via the angiotensin-converting enzyme 2 receptor, which is essential for adequate functioning of the circulatory system. COVID-19 can be divided into three stages: early infection, pulmonary involvement (IIa without or IIb with hypoxia), and systemic hyperinflammation (cytokine storm) [1]. The last stage, similarly to myocardial infarction (MI), is associated with uncontrolled propagation of inflammation mediated by molecules including IL-2, IL-6, IL-7, granulocyte colony-stimulating factor, macrophage inflammatory protein 1-α, tumor necrosis factor-α, C-reactive protein, ferritin, and D-dimer [2].

Several studies have demonstrated that COVID-19 affects cardiovascular system function. COVID-19 alone has been shown to increase the risk of thrombotic events, including MI, acute limb ischemia, abdominal and thoracic aortic thrombosis, mesenteric ischemia, pulmonary embolism, stroke, and disseminated intravascular coagulation. COVID-19 can cause heart muscle inflammation and injury in an MI-independent manner. At the health care system level, COVID-19 may perturb acute coronary syndrome hospitalization pathways, thus decreasing the rate of MI hospitalizations [3,4,5,6,7,8,9] worldwide, delaying patients’ contact with medical services [10,11,12] and emergency department arrival times of patients with suspected acute coronary syndrome [13] and increasing the door-to-balloon time [14,15,16], scar size (as confirmed with cardiac magnetic resonance) [17], and rate of mechanical complications. Moreover, during the COVID-19 pandemic, the prolongation of time from the first medical contact to revascularization was revealed in a group of patients with STEMI and out-of-hospital cardiac arrest [18]. At the procedural level, COVID-19 is a factor increasing the risk of aspiration thrombectomy [19]. Some studies have shown that SARS-CoV-2 infection alone can be classified as a risk factor/trigger of MI [20]. Regardless of the above associations, the results of studies assessing the global influence of the COVID-19 pandemic on the mortality of patients with MI have been inconsistent: some studies have indicated elevated in-hospital mortality [21,22,23,24,25,26], whereas others have not [9,27]. Consequently, this multicenter retrospective study with a control group was designed to assess the influence of SARS-CoV-2 infection on treatments and outcomes in patients with MI.

## 2. Materials and Methods

### 2.1. Study Group

Among 6490 patients with MI treated in 11 American Heart of Poland Cardiology Departments from 4 March 2020 (the date of the first identified COVID-19 case in Poland) until 4 March 2022 (spanning the first 2 years of the COVID-19 pandemic), 165 patients with COVID-19 were identified. The patients were considered COVID-19-positive on the basis of PCR test results. Hospitalization histories were analyzed retrospectively. The minimum follow-up time was 1 month, and the maximum follow-up time was 6 months. All inclusion criteria were based on the participants’ medical records. Patients were recruited according to the following inclusion criteria:

- Confirmed MI, according to the ESC Fourth Universal Definition of Myocardial Infarction;
- Active SARS-CoV-2 infection confirmed during hospitalization by PCR testing;
- Age > 18 years.

All exclusion criteria were based on the participants’ medical records. The exclusion criteria were as follows:

- Any coexisting disease potentially limiting lifetime during observation (e.g., any end-stage organ failure or end-stage cancer);
- Minimum follow-up duration not met;
- Lack of primary clinical data necessary for matching, e.g., glomerular filtration rate (GFR) or ejection fraction (EF).

Thirty-six patients were excluded from this study on the basis of the following criteria: end-stage disease (two patients, 5.5%), minimum follow-up duration not met (fourteen patients, 38.9%), and insufficient data to identify matching patients (twenty patients, 55.5%). Ethical approval was not obtained because all data were collected during standard care procedures.

### 2.2. Study Objectives and Endpoints

The primary objective of this study was to assess the influence of SARS-CoV-2 infection on in-hospital mortality in patients treated for MI and COVID-19 simultaneously. The secondary objectives of this study were as follows: assessment of the influence of SARS-CoV-2 infection on out-of-hospital mortality among patients previously treated for MI and COVID-19 simultaneously and assessment of the influence of basic clinical parameters on in-hospital mortality among patients treated for MI and COVID-19 simultaneously. The primary endpoint was defined as the time to in-hospital death. The secondary endpoint was defined as the time to out-of-hospital death.

### 2.3. Methods

**Figure 1:**
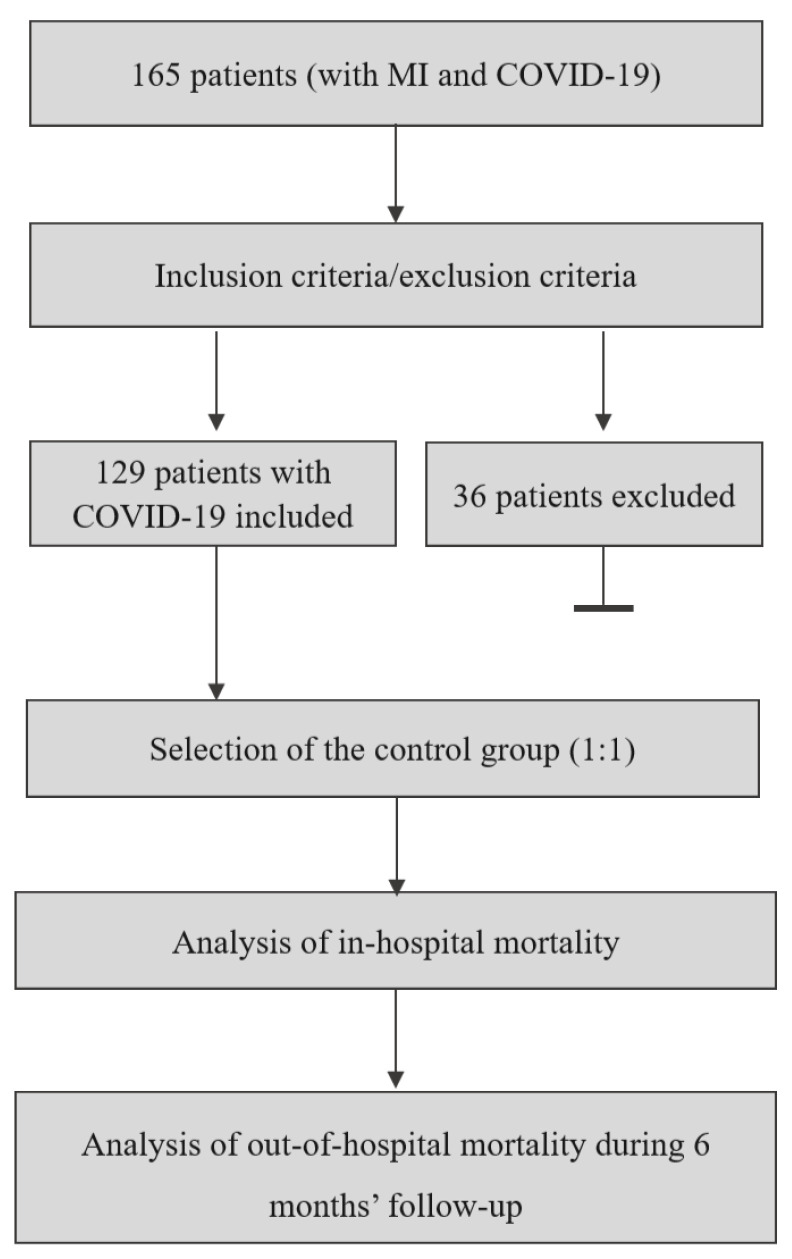
Study design. COVID-19: coronavirus disease 2019, MI: myocardial infarction.

The study scheme is presented in Figure 1.

All clinical data were retrospectively obtained from the American Heart of Poland patient database. After the creation of the study group, the control group was created by selecting 1:1 comparable patients in terms of age, sex, diabetes mellitus (DM) presence, MI type, initial ejection fraction, and initial GFR. The accuracy of matching was additionally verified using propensity score. Then, subsequent analysis was conducted. To assess the primary objective of this study, we compared overall in-hospital mortality between the study group and control group. The analysis was extended to compare in-hospital mortality in subgroups of patients according to MI type (ST-elevation MI (STEMI) or non-ST-elevation MI (NSTEMI)), sex (male or female), presence of heart failure with reduced ejection fraction (EFrEF; HFrEF subgroup and no HFrEF subgroup), presence of DM, and need for intensive care unit (ICU) hospitalization. In COVID-19 subgroups, additional analyses compared in-hospital mortality in STEMI vs. NSTEMI and ICU patients vs. the rest of the COVID-19 group. The analysis was extended to outcome predictors in the overall group (including both the study group and the control group). Finally, out-of-hospital mortality was assessed in the study group vs. the controls. To achieve comparable overall observation time (including both in-hospital and out-of-hospital observations), we extended the follow-up to 6 months. In-hospital mortality and overall mortality were analyzed.

### 2.4. Statistical Analysis

Statistical analysis was performed in PQStata version 1.8.4 (PQStat Software) and MedCalc version 20.106 (MedCalc Software). The obtained data are presented as percentages, means and standard deviations (for parametric data), or medians with interquartile ranges (for nonparametric data). The following statistical tests were applied: Shapiro–Wilk test (for normality assessment), Student’s *t*-test (for difference analysis of data with a normal distribution), Mann–Whitney U test (for difference analysis of data with a nonparametric distribution), and chi-square test (for proportion difference analysis). Propensity score matching was used to create the control group. Kaplan–Meier plots were used for survival analysis. Cox proportional hazard models were used to analyze the influence of selected risk factors on survival. Results were considered statistically significant if *p* < 0.05 and highly statistically significant if *p* < 0.001.

### 2.5. Sample Size Determination

The minimum total sample size (for both groups together) for the comparison of two proportions with type I error of 0.05 and type II error of 0.2 was estimated to be 68. For Cox multivariable proportional hazard regression, the minimum number of cases to be included in this study was established to be 204.

### 2.6. Rationale for Study Design

This article provides an overview of the influence of SARS-CoV-2 infection on the mortality of patients simultaneously being treated for MI. In contrast to most reports, the observation time in this study was prolonged for as many as 6 months to obtain both in-hospital and out-of-hospital observations. Whereas most prior articles have focused on patients with STEMI, patients with both STEMI and NSTEMI were included herein. The presented data provide rare evidence that SARS-CoV-2 infection influences the in-hospital outcomes of patients with NSTEMI. Additionally, the roles of COVID-19 and respiratory failure as outcome predictors among patients treated for MI were assessed.

## 3. Results

### 3.1. Study Group

A total of 165 patients treated for MI were COVID-19-positive. SARS-CoV-2 infection was present in 25.4 of every 1000 patients with MI (2.5%). Of all patients with COVID-19, 129 met the inclusion criteria and were included in this study. The study group ultimately consisted of 92 men (71.32%), 37 women (28.68%), 63 patients with STEMI (48.84%), and 66 patients with NSTEMI (51.16%). The control group consisted of 129 patients with MI without COVID-19. The groups were comparable in age, sex, MI type, coexisting DM, EF, and initial GFR. The demographic and clinical characteristics of the study group and the control group are presented in Table 1, clinical characteristics section.

The procedural characteristics are presented in Table 1, procedural characteristics section. No differences between the study and control groups were observed in qualification for percutaneous coronary intervention, coronary artery bypass grafting (CABG), optimal medical treatment, overall length of implanted stents, and the number of treated vessels. The hospitalization characteristics are presented in Table 1, hospitalization characteristics section. Subsequent differences in hospitalizations were identified. Cardiac shock was observed in eighteen (13.95%) patients in the study group and eight (6.2%) patients in the control group (*p* = 0.04). Respiratory failure requiring mechanical ventilation was observed in 23 (17.83%) patients who were finally transferred to the ICU in the study group and no patients in the control group (*p* < 0.001). Bleeding requiring transfusion was observed in five (3.88%) patients in the study group and no patients in the control group (*p* = 0.02).

### 3.2. In-Hospital Mortality and Survival Analysis

**Figure 2:**
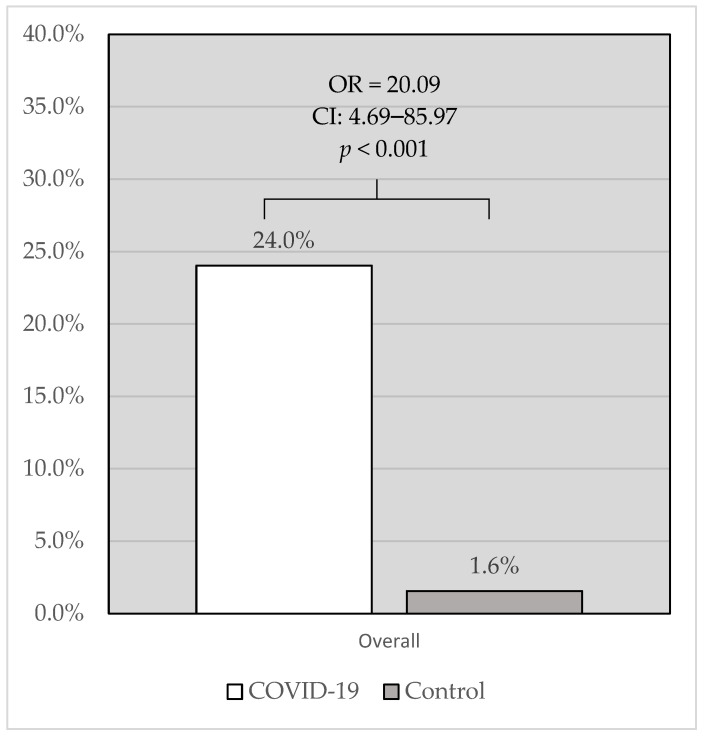
Comparison of overall in-hospital mortality in patients with COVID-19 vs. controls. COVID-19: coronavirus disease 2019.

A total of thirty-one (24%) patients died in the study group, whereas two (1.6%) patients died in the control group (OR = 20.09; CI: 4.69–85.97; *p* < 0.001; Figure 2).

**Figure 3:**
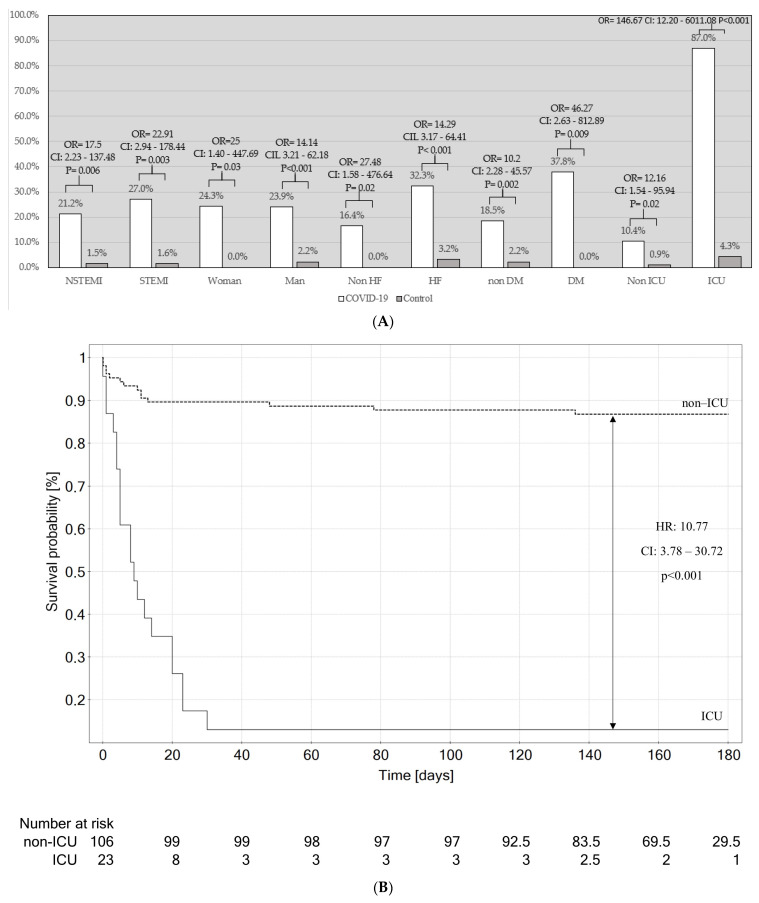
Mortality analysis in subgroups of patients. DM: diabetes mellitus; HF: heart failure; ICU: intensive care. (**A**) Comparison of in-hospital mortality in COVID-19 subgroups vs. control subgroups. (**B**) Kaplan–Meier analysis of 6-month overall mortality in the COVID-19 intensive care unit patient subgroup vs. COVID-19 non-intensive care unit patient subgroup.

When all patients were included, regardless of the availability of clinical data to identify matching patients (142 patients), the overall in-hospital mortality in the study group was even higher: 41 (28.9%) patients died. Similar results were observed in all analyzed patient subgroups (Figure 3A).

The greatest increase in mortality (87%) was observed in the subgroup of patients requiring ICU hospitalization (87.0% vs. 4.3%; OR = 146.67; CI: 12.20–6011.08; *p* < 0.001; Figure 3). The obtained data were verified with both univariable and multivariable Cox regression models, thus confirming the significant influence of SARS-CoV-2 infection on in-hospital outcomes (HR: 9.10; CI: 2.12–39.00; *p* = 0.003; Table 2). Moreover, the results indicated the value of acute respiratory failure development (treated with mechanical ventilation) in predicting the fatal outcome, with high statistical significance (HR: 6.80; CI: 3.32–13.94; *p* < 0.001).

Additional analysis was conducted in the COVID-19 subgroup (STEMI vs. NSTEMI and ICU vs. non-ICU). The mortality in the STEMI and NSTEMI COVID-19 subgroups was comparable: 14 (21.12%) patients died in the NSTEMI subgroup, and 17 (26.98%) patients died in the STEMI subgroup (OR: 1.3; CI: 0.56–3.37; *p* = 0.45). In addition, the mortality was comparable in patients with COVID-19 between the first and second years of the pandemic (OR: 1.36; CI: 0.53–3.39; *p* = 0.46). The lack of influence of pandemic year was also confirmed with univariable Cox regression (HR: 0.86; CI: 0.42–1.79; *p* = 0.75). The in-hospital mortality in the ICU subgroup was higher than that in the non-ICU subgroup, at 20 patients (86.96%) vs. 11 patients (10.38%; OR = 54.16; CI: 13.27–327.50; *p* < 0.001). Figure 3B presents the overall (including in-hospital and out-of-hospital) mortality in the COVID-19 intensive care unit patient subgroup vs. COVID-19 non-intensive care unit patient subgroup.

**Figure 4:**
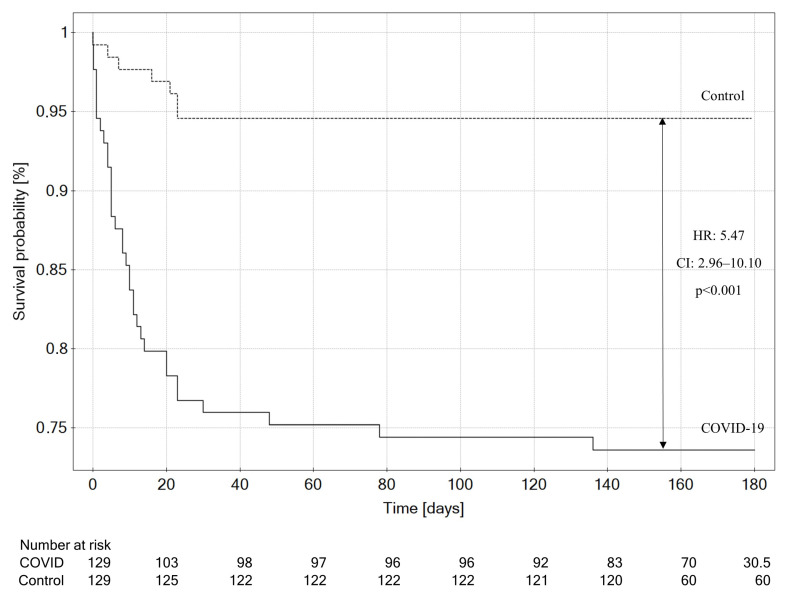
Kaplan–Meier analysis of 6-month overall mortality, comparing the COVID-19 group and controls. COVID-19: coronavirus disease 2019.

During out-of-hospital observation, three (3.06%) patients died in the study group, and five patients (3.94%) died in the control group (OR: 0.77; CI: 0.11–4.07; *p* = 0.73). During the overall observation period (including both in-hospital and out-of-hospital data), thirty-four (26.4%) patients died in the study group, whereas eight (6.2%) patients died in the control group (HR: 5.47; CI: 2.96–10.10; *p* < 0.001; Figure 4).

## 4. Discussion

In-hospital mortality can be as high as 7% in patients with STEMI and 4.9% in patients with NSTEMI [28]. On the basis of WHO data, the COVID-19 mortality in the general population is approximately 3.4% [29] and ranges from 0.1% to 5.2%. In contrast, the seasonal influenza mortality has been estimated to be <1%. Among hospitalized patients with COVID-19, the mortality has been reported to be approximately 21% [30]. The frequency of COVID-19 in patients treated for STEMI reached up to 1% in epidemiological studies [31,32]. In our study it was 2.5%. The obtained result may be a positive consequence of performed cyclical PCR screening tests as a standard of care in treated patients. The mortality in patients with COVID-19 and coexisting MI varies from 10% to 76.6%. Lower mortality rates have often been observed in earlier studies. Perioperatively, the mortality rate reached 1.29% in patients with STEMI treated for COVID-19 [19]. In later, more complex studies covering the full period of hospitalization, the mortality has reached approximately 30%, regardless of geographic region. Most of the obtained results have been statistically significant. Among the relevant studies, only Choudry et al. observed a numerical but not statistically significant difference in the mortality of COVID-19 patients. In addition, Zajac et al. did not observe a statistical difference in mortality. However, in that study, the observation time was limited to the procedure [19,32,33,34,35,36,37,38,39,40,41]. Among cited studies, only Kite et al. and Markson et al. assessed outcomes among patients with NSTEMI [37]. In their studies, the mortality in the STEMI group was higher (6.6% vs. 21.1% and 9.1% vs. 17.2%, respectively), in contrast to the findings in our study, in which the mortality was similar between NSTEMI and STEMI.

The mortality rate of patients with HF and COVID-19 can reach 45% [42,43,44,45,46]. Moreover, hospital outcomes depend on the initial EF in patients with COVID-19 [47]. Various studies and meta-analyses have demonstrated that DM strongly affects COVID-19 in-hospital mortality, which has been found to reach 22.14% [48]. In patients requiring invasive mechanical ventilation or ICU hospitalization, the mortality ranges from 9.9% to 78.4% and commonly exceeds 30% [49,50].

Our risk factor analysis results slightly differed from those reported by other authors. In the present study, COVID-19 and respiratory failure were identified as the main risk factors for fatal outcomes. Additionally, the EF appeared to slightly modify the mortality risk, which is a common result observed in patients with MI. These factors were not included in other analyses. In a study by Kazirod-Wolski et al., age and STEMI type were identified as predictors of periprocedural fatal outcomes, whereas our analysis did not indicate similar results. The difference between studies may be a consequence of different observation times (assessment during the full hospitalization time herein vs. the periprocedural period in other studies) [51].

In various studies, fluctuations in mortality have been observed among subsequent pandemic waves. Some studies have indicated an increasing mortality trend [52], whereas others have documented a decreasing mortality trend [53,54,55]. In the present study, no statistical differences in mortality were observed between the first and the second years of the pandemic. Moreover, the pandemic year did not reach statistical significance in the regression model. These results indicate that SARS-CoV-2 infection can strongly and permanently influence the outcomes of patients with MI. The number of patients increased between the first and second years of the pandemic, in agreement with other authors’ observations [8].

The obtained data yielded several conclusions. The mortality rate in the overall COVID-19 group and subgroups was higher than that in the control group. These results are comparable to those obtained by other researchers. The mortality rate in the DM subgroup was slightly higher than those reported in other studies [48,56,57], possibly because of the elevated overall mortality of patients with DM treated for MI [58,59]. The mortality in HF patients with COVID-19 can reach 50% [43,60,61,62,63]. In the present study, the mortality rate in the HF subgroup was slightly lower. However, in studies assessing the influence of COVID-19 on in-hospital outcomes in patients with HF, the EF was often unknown, whereas the present study relied on echocardiographic assessment during hospitalization. Potential differences in EF may be a reason for the observed incompatibility in in-hospital outcomes. Moreover, comparison between HF groups may be difficult because of their heterogeneity, including different etiologies and a variety of possible coexisting diseases. In the present study, the respiratory failure frequency was higher in the study group than in the control group and appeared to be the main reason for the increased mortality. We also observed that the cardiac shock frequency was higher in the study group, possibly as a consequence of COVID-19-mediated heart muscle injury. Cardiac shock also contributed to elevated mortality in the study group. Similar observations have been reported by Kite et al. [37].

In contrast to other studies focusing mainly on patients with STEMI, this study examined a heterogeneous group including both patients with STEMI and NSTEMI. Most previous studies have excluded patients with NSTEMI. The difference in mortality between patients with STEMI and NSTEMI has been considered only by Kite et al. [37], who have observed greater mortality in patients with STEMI than NSTEMI. In contrast, our results indicated comparable mortality between STEMI and NSTEMI groups. Moreover, whereas other studies have focused only on in-hospital observations, the present research was extended to 6 months, including out-of-hospital follow-up. On the basis of the presented data, the higher morality observed in patients with COVID-19 was a consequence of in-hospital mortality.

## 5. Study Limitations

Before the statistical analysis, 36 patients were excluded from this study (mainly because of insufficient clinical data), and their outcomes were difficult to compare. The results might have been biased because of the relatively small size of the study group. The study did not reach sufficient statistical power to include the Grace score in the Cox model analysis. Multiple comparisons between the study group and the control group were not conducted because most of the obtained data did not reach normal distribution.

## 6. Conclusions

On the basis of the presented results, we reached the following conclusions. SARS-CoV-2 infection significantly affects the in-hospital outcomes of patients with both COVID-19 and MI. An elevated frequency of both respiratory failure and cardiac shock explained the elevated mortality observed among patients with COVID-19 treated for MI.

## Figures and Tables

**Table 1:** Basic clinical, procedural, and hospitalization characteristics of the COVID-19 group vs. controls.

	COVID-19 (*n* = 129)	Control (n = 129)	*p*
Clinical Characteristics			
Men, n (%)	92 (71.32%)	92 (71.32%)	
Women, n (%)	37 (28.68%)	37 (28.68%)	
NSTEMI, n (%)	66 (51.16%)	66 (51.16%)	
STEMI, n (%)	63 (48.84%)	63 (48.84%)	
Age, years	67 (61–75)	67 (60–75)	0.52
EF, %	45 (35–50)	45 (35–50)	0.93
GFR, mL/min	75.8 (53.5–94.5)	80.14 (55.2–95.7)	0.56
Diabetes, n (%)	37 (28.68%)	37 (28.68%)	
Hypertension, n (%)	86 (66.67%)	100 (77.52%)	0.05
Hyperlipidemia, n (%)	56 (43.41%)	76 (58.91%)	0.01
Smoking, n (%)	17 (13.18%)	33 (25.58%)	0.01
Peripheral arterial disease, n (%)	2 (1.55%)	13 (10.08%)	0.004
Obesity, n (%)	16 (12.4%)	16 (12.4%)	
Previous stroke, n (%)	8 (6.2%)	13 (10.08%)	0.19
Atrial fibrillation, n (%)	23 (17.83%)	19 (14.73%)	0.50
Myocardial infarction, n (%)	25 (19.38%)	29 (22.48%)	0.54
Previous PTCA, n (%)	20 (15.5%)	26 (20.16%)	0.33
Previous CABG, n (%)	8 (6.2%)	5 (3.88%)	0.39
Procedural characteristics			
Coronarography, n (%)	125 (96.9%)	128 (99.22%)	0.18
OMT, n (%)	4 (3.1%)	1 (0.78%)	0.18
CABG qualified, n (%)	8 (6.2%)	5 (3.88%)	0.39
PTCA, n (%)	107 (82.95%)	112 (86.82%)	0.85
Reached TIMI 3, n (%)	91 (85.05%)	105 (93.75%)	0.02
POBA, n (%)	(0%)	(0%)	
MVD, n (%)	35 (27.13%)	20 (15.5%)	0.02
LM, n (%)	5 (3.88%)	6 (4.65%)	0.76
LAD, n (%)	41 (31.78%)	49 (37.98%)	0.30
D1, n (%)	4 (3.1%)	4 (3.1%)	
CX, n (%)	11 (8.53%)	16 (12.4%)	0.31
OM, n (%)	4 (3.1%)	9 (6.98%)	0.15
RCA, n (%)	25 (19.38%)	29 (22.48%)	0.54
PDA, n (%)	0 (0%)	2 (1.55%)	0.16
IM, n (%)	1 (0.78%)	2 (1.55%)	0.56
Bridge, n (%)	2 (1.55%)	3 (2.33%)	0.65
Stent length, mm	32 (20–51.5)	25.5 (18–40)	0.11
Multisteps, n (%)	4 (3.1%)	12 (9.3%)	0.04
Hospitalization characteristics			
Cardiogenic shock, n (%)	18 (13.95%)	8 (6.2%)	0.04
Pulmonary edema, n (%)	9 (6.98%)	23 (17.83%)	0.008
Respiratory failure, n (%)	23 (17.83%)	0 (0%)	<0.001
Contrast-induced nephropathy, n (%)	12 (9.3%)	5 (3.88%)	0.08
Stroke, n (%)	1 (0.78%)	0 (0%)	0.32
Bleeding requiring transfusion, n (%)	5 (3.88%)	0 (0%)	0.02
Hospitalization time (days)	10 (4–14)	4 (3–5)	<0.001
Time to death (days)	8 (3.25–13.75)	16 (6–22)	0.28
Transfer to another ward frequency, n (%)	72 (55.81%)	10 (7.75%)	<0.001

CABG: coronary artery bypass graft; CX: circumflex artery; D1: diagonal branches of the left anterior descending artery; EF: ejection fraction; GFR: glomerular filtration rate; IM: intermediate artery; LAD: left anterior descending artery; LM: left main coronary artery; MVD: multivessel coronary artery disease; NSTEMI: non-ST-elevation myocardial infarction; OM: obtuse marginal artery; OMT: optimal medical therapy; PDA: posterior descending artery; POBA: balloon angioplasty; PTCA: percutaneous transluminal coronary angioplasty; RCA: right coronary artery; STEMI: ST-elevation myocardial infarction; TIMI3: thrombolysis in myocardial infarction.

**Table 2:** Cox univariable and multivariable regression analyses for predictors of all-cause mortality.

	Univariable Cox Regression				Multivariable Cox Regression			
Parameter	HR	−95% CI	+95% CI	*p*	HR	−95% CI	+95% CI	*p*
COVID-19	7.45	1.74	32.01	0.007	9.10	2.12	39.00	0.003
Acute respiratory failure with mechanical ventilation	6.73	3.29	13.78	<0.001	6.80	3.32	13.94	<0.001
Sex	1.20	0.56	2.61	0.64				
Diabetes	1.70	0.85	3.39	0.13				
GFR, mL/min	0.99	0.98	1.00	0.076				
EF, %	0.97	0.94	0.99	0.04	0.96	0.93	0.99	0.013
Age, years	1.01	0.98	1.04	0.40				
MI type (STEMI vs. NSTEMI)	1.29	0.64	2.58	0.47				

COVID-19: coronavirus disease 2019; EF: ejection fraction; GFR: glomerular filtration rate; MI: myocardial infarction.

## Data Availability

Derived data supporting the findings of this study are available from the corresponding author on request.
